# One Complete and Seven Draft Genome Sequences of Subdivision 1 and 3 *Acidobacteria* Isolated from Soil

**DOI:** 10.1128/MRA.01087-19

**Published:** 2020-01-30

**Authors:** Stephanie A. Eichorst, Daniela Trojan, Marcel Huntemann, Alicia Clum, Manoj Pillay, Krishnaveni Palaniappan, Neha Varghese, Natalia Mikhailova, Dimitrios Stamatis, T. B. K. Reddy, Chris Daum, Lynne A. Goodwin, Nicole Shapiro, Natalia Ivanova, Nikos Kyrpides, Tanja Woyke, Dagmar Woebken

**Affiliations:** a Division of Microbial Ecology, Department of Microbiology and Ecosystem Science, Centre for Microbiology and Environmental Systems Science, University of Vienna, Vienna, Austria; b Department of Energy, Joint Genome Institute, Berkeley, California, USA; c Materials Science and Technology, Engineered Materials, Los Alamos National Laboratory, Los Alamos, New Mexico, USA; Indiana University, Bloomington

## Abstract

We report eight genomes from representatives of the phylum *Acidobacteria* subdivisions 1 and 3, isolated from soils. The genome sizes range from 4.9 to 6.7 Mb. Genomic analysis reveals putative genes for low- and high-affinity respiratory oxygen reductases, high-affinity hydrogenases, and the capacity to use a diverse collection of carbohydrates.

## ANNOUNCEMENT

The *Acidobacteria* constitute a large, diverse, and phylogenetically distinct phylum with currently 26 defined subdivisions based on the 16S rRNA gene phylogeny (1). Typically, members of subdivisions 1, 2, 3, 4, 5, and 6 populate terrestrial environments at a high relative sequence abundance based on rRNA gene libraries (as high as 20 to 40% of the total bacterial community) (2, 3). Phylogenetically representative acidobacterium strains were chosen for genome sequencing, which were isolated from soils (meadow grassland, agricultural, or peat bog) (4–7). More specifically, six strains in subdivision 1 (Terriglobus roseus KBS 63, *Acidobacteriaceae* bacterium strain KBS 83, *Acidobacteriaceae* bacterium strain KBS 89, *Acidobacteriaceae* bacterium strain KBS 146, *Terriglobus* sp. strain TAA 43, and *Acidobacteriaceae* bacterium strain TAA 166) and two strains in subdivision 3 (*Acidobacteria* bacterium strain KBS 96 and Bryobacter aggregatus MPL3) were sequenced to better populate the phylogenetic branches in the phylum *Acidobacteria* with genomic representation.

Strains were grown on either a modified minimal medium or R2 medium as described previously (5–7). Genomic DNA (gDNA) was isolated using a modified cetyltrimethylammonium bromide (CTAB) DNA extraction protocol as recommended by the Department of Energy (DOE) Joint Genome Institute (JGI). Details on the isolation and growth conditions along with the sequencing can be found in Table 1. The genomes across all strains ranged in size from 4.9 to 6.7 Mb (Table 1), which is similar to the size range of the previously sequenced acidobacterial strains (8, 9). The G+C content ranged from 57 to 60 mol% (Table 1). Although many of these genomes are considered to be permanent drafts (Table 1), CheckM analysis indicated that they are ca. >95% complete (10).

**TABLE 1:** Isolation conditions, library preparation information, genome sequencing statistics, and accession numbers for the described acidobacterial genomes

Strain	Isolation source; yr	Isolation and growth conditions (reference)	Library prepn	No. of reads (technology)	Avg read length (bp)	Assembler (reference)	No. of contigs (no. of scaffolds)	*N*/*L*_50_ of:		Avg genome coverage (×)	Genome size (Mb)	G+C content (mol%)	Assembly level	GenBank accession no.	SRA accession no.
								Scaffolds	Contigs						
KBS 63	Kellogg Biological Station, Hickory Corners, MI, USA, grassland soil (treatment 8); 2003	VSB-7 mix organic C substrates in CO_2_-enriched air (5)	454 paired-end library;^*a*^ Illumina std. shotgun library^*b*^	69,481,460 (Illumina Solexa); 514,324 (454 paired end)	76 (Illumina Solexa); 151 (454 paired end)	Newbler v2.3-Prerelease 6/30/2009, Velvet v1.0.13 (13)	33 (2)	1/5.2 Mb	1/5.2 Mb	1083.1 (Illumina Solexa); 8.5 (454 paired end)	5.23	60.3	Complete	CP003379	SRS1568612
KBS 83	Kellogg Biological Station, Hickory Corners, MI, USA, agricultural soil (treatment 1); 2006	VL55 mix of plant polymeric C in air (7)	Illumina std. shotgun library^*b*^	12,985,884 (Illumina HiSeq 2000)	2 × 150	Velvet v1.1.04 (13), wgsim^*e*^, Allpaths-LG v r41043 (14)	25 (25)	5/504.7 kb	5/504.7 kb	390	6.25	59.2	Permanent draft	ARMD00000000	SRS844143
KBS 89	Kellogg Biological Station, Hickory Corners, MI, USA, grassland soil (treatment 8); 2002	VSB-6.8 mix organic C substrates, acyl homoserine lactones in CO_2_-enriched air (4, 5)	Illumina std. shotgun library^*b*^	12,475,762 (Illumina HiSeq 2000)	2 × 150	Velvet v1.1.04 (13), wgsim^*e*^, Allpaths-LG v r41043 (14)	14 (14)	3/943.6 kb	3/943.6 kb	122.6	6.01	57.6	Permanent draft	ARME00000000	SRS844144
KBS 146	Kellogg Biological Station, Hickory Corners, MI, USA, grassland soil (treatment 8); 2006	VL55 mix of organic C substrates, CO_2_-enriched hypoxia (11)	PacBio SMRTbell library^*d*^	185,131 (PacBio RS platform)	3,493 ± 2,894	HGAP v2.0.0 (15)	2 (2)	1/5.0 Mb	1/5.0 Mb	64.1	5.00	56.7	Permanent draft	JHVA00000000	SRS1534005
TAA 43	Hindgut of Reticulitermes flavipes (Kollar) (*Phinotermitidae*), Dansville, MI, USA; 2002	VSB-7 yeast extract and peptone in CO_2_-enriched air (4, 5)	Illumina std. shotgun library^*b*^	13,649,630 (Illumina std. paired end, Illumina HiSeq 2000)	2 × 151	Velvet v1.2.07 (13), wgsim^*e*^, Allpaths-LG v r46652 (14)	7 (7)	1/3.5 Mb	1/3.5 Mb	302.0	4.95	56.7	Permanent draft	JUGR00000000	SRS1366045
TAA 166	Hindgut of *R. flavipes* (Kollar) (*Phinotermitidae*), Dansville, MI, USA; 2002	VSB-7 yeast extract and peptone in CO_2_-enriched air (4, 5)	Illumina std. shotgun library^*b*^ and long-insert mate pair library^*c*^; PacBio SMRTbell library^*d*^	40,920,398 (Illumina CLIP paired end, Illumina HiSeq 2000); 15,055,388 (Illumina std. paired end, Illumina HiSeq 2000); 233,258 (PacBio RS platform)	2 × 90; 2 × 150; 1 × 2259	AllpathsLG vr42328 (14)	3 (3)	1/4.7 Mb	1/4.7 Mb	973.9 (Illumina); 85.4 (PacBio)	6.14	58.8	Permanent draft	ATWD00000000	SRS438888
KBS 96	Kellogg Biological Station, Hickory Corners, MI, USA, agricultural soil (treatment 1); 2006	VL55 mix of plant polymeric C in air (7)	Illumina std. shotgun library^*b*^ and long-insert mate pair library^*c*^	14,485,381 (Illumina std. paired end, HiSeq 2000); 58,296,769 (Illumina CLIP paired end, Illumina HiSeq 2000)	2 × 150; 2 × 89	Allpaths-LG v r41043 (14)	13 (2)	1/6.7 Mb	2/1.6 Mb	1,098.6	6.69	57.2	Permanent draft	ARMF00000000	SRS438892
MPL3	Acidic *Sphagnum* peat bog, Bakchar, Tomsk region, West Siberia; 2004	Biofilm-mediated enrichment approach (6, 12)	PacBio SMRTbell library^*d*^	208,346 (PacBio RS platform)	3,087 ± 2,325	HGAP v2.0.0 (15)	4 (4)	1/4.3 Mb	1/4.3 Mb	176.8	5.75	57.0	Permanent draft	JNIF00000000	SRS1520682

a 454 Titanium, paired ends, 8 kb; 15 μg genomic DNA are sheared by the Hydroshear to ~8-kb size fragments. The sheared samples are then gel selected for the 8-kb bands, purified, and ligated to the 42-bp loxP linkers on either end. These loxP linkers are labeled by biotin. The loxP linker-ligated fragments are then circularized by the Cre recombinase. As a result, the ends of 20-kb fragments are brought together and bridged by a single loxP linker. These circular DNAs are further sheared to 500-bp fragments, and the fragments carrying the loxP linkers are recovered by the Streptavidin-coated magnetic beads. Consequently, the loxP linker-containing fragments are ligated to the 454 Titanium adapters A and B in the same way that the shotgun libraries are created. The 454 library fragments are then clonally amplified in bulk by capturing them through hybridization on microparticle beads and subjecting them to emulsion-based PCR. This results in beads that are covered with millions of copies of a single DNA fragment (range, 400--800 bp), where each bead contains a different clonally amplified library fragment. After amplification, the beads are recovered from the emulsions and are loaded into the wells of a PicoTiterPlate (PTP) device such that wells contain single DNA beads. The PTP device is then inserted into the 454 genome sequencer FLX-Titanium instrument for sequencing where sequencing reagents are sequentially flowed over the PTP wells. Each incorporation of a nucleotide complementary to the template strand results in a chemiluminescent light signal that is recorded by a camera, and the sequence of the DNA fragments is determined. This sequencing-by-synthesis method is known as pyrosequencing.

b Illumina regular fragment, 300 bp; 100 ng of DNA was sheared to 300 bp using the Covaris LE220 instrument and size selected using solid-phase reversible immobilization (SPRI) beads (Beckman Coulter). The fragments were treated with end repair, A-tailing, and ligation of Illumina-compatible adapters (IDT, Inc.) using the KAPA-Illumina library creation kit (Kapa Biosystems). The prepared libraries were quantified using Kapa Biosystem’s next-generation sequencing library quantitative PCR (qPCR) kit and run on a Roche LightCycler 480 real-time PCR instrument. The quantified libraries were then multiplexed with other libraries, and the pool of libraries was then prepared for sequencing on the Illumina HiSeq or the Illumina GAIIx (KBS 63) sequencing platform utilizing a TruSeq paired-end cluster kit v3 or a paired-end cluster generation kit, v4 (KBS 63) and Illumina’s cBot instrument to generate a clustered flow cell for sequencing. Sequencing of the flow cell was performed on the Illumina HiSeq 2000 sequencer using a TruSeq SBS sequencing kit v3 following a 2 × 150 indexed run recipe and on the Illumina GAIIx sequencer using SBS sequencing kits, v4, following a 2 × 76 run recipe (KBS 63).

c Illumina regular long mate pair, 8 kb, Cre-Lox random shear mate pair sequences; 5 μg of DNA was sheared using = Covaris g-TUBEs and was gel size selected for 8 kb. The sheared DNA was treated with end repair and ligated with biotinylated adapters containing loxP. The adapter-ligated DNA fragments were circularized via recombination with a Cre excision reaction (NEB). The circularized DNA templates were then randomly sheared using the Covaris LE220 instrument. The sheared fragments were treated with end repair and A-tailing using the KAPA-Illumina library creation kit (Kapa Biosystems) followed by immobilization of mate pair fragments on streptavidin beads (Invitrogen). Illumina-compatible adapters (IDT, Inc.) were ligated to the mate pair fragments, and 14 cycles of PCR were used to enrich for the final library (Kapa Biosystems). The prepared libraries were quantified using Kapa Biosystem’s next-generation sequencing library qPCR kit and run on a Roche LightCycler 480 real-time PCR instrument. The quantified libraries were then multiplexed with other libraries, and the pool of libraries was then prepared for sequencing on the Illumina HiSeq sequencing platform utilizing a TruSeq paired-end cluster kit v3 and Illumina’s cBot instrument to generate a clustered flow cell for sequencing. Sequencing of the flow cell was performed on the Illumina HiSeq 2000 sequencer using a TruSeq SBS sequencing kit v3 following a 2 × 100 indexed run recipe.

d PacBio >10-kb libraries with AMPure bead size selection; unamplified libraries were generated using the Pacific Biosciences standard template preparation protocol for creating >10-kb libraries. A total of 5 μg of genomic DNA was used to generate each library, and the DNA was sheared using Covaris g-TUBEs to generate sheared fragments >10 kb in length. The sheared DNA fragments were then prepared using Pacific Biosciences SMRTbell template preparation kit v1.0, where the fragments were treated with DNA damage repair, had their ends repaired so that they were blunt ended, and were 5′ phosphorylated. Pacific Biosciences hairpin adapters were then ligated to the fragments to create the SMRTbell template for sequencing. The SMRTbell templates were then purified using exonuclease treatments and size selected using AMPure PB beads. Sequencing primer was then annealed to the SMRTbell templates, and version XL sequencing polymerase was bound to them. The prepared SMRTbell template libraries were then sequenced on a Pacific Biosciences RS II sequencer using version C2 chemistry and 2-h sequencing movie run times.

e https://github.com/lh3/wgsim.

The genomes of KBS 63 and KBS 96 harbored the catalytic subunit for the high-affinity *cbb_3_*-type cytochrome *c* oxidase (heme-copper-oxygen reductase [HCO] type C, EC 1.9.3.1, KEGG orthology [KO] number K00404), while the genomes of KBS 146 and TAA 166 harbored the high-affinity cytochrome *bd*-type quinol oxidase (EC 7.1.1, KO number K00425) (10). All strains harbored at least one homologue of the low-affinity terminal oxidase HCO type A (EC 1.9.3.1, KO number K02274) (10). Numerous glycoside hydrolase (GH) families were detected across the genomes, with GH109, GH74, and GH13 being some of the most prevalent (10). The genomes of KBS 83 and KBS 96 harbored a high-affinity group 1h hydrogenase (EC 1.12.99.6, KO number K06281), presumably giving them the potential to scavenge atmospheric concentrations of hydrogen during periods of starvation (10). The addition of these genomes in the public databases will help to further our understanding of this prevalent and diverse phylum.

### Data availability.

The genome sequences are available in GenBank; accession numbers can be found in Table 1.
